# One‐year follow‐up after fractionated ultra‐high‐dose‐rate FLASH radiotherapy in patient with extramammary Paget disease of the scrotum

**DOI:** 10.1002/pro6.70045

**Published:** 2025-12-26

**Authors:** Hui Luo, Chengliang Yang, Ronghu Mao, Leijie Ma, Hongchang Lei, Peng Chen, Yanping Zhang, Meng Xu, Chao Wang, Yiwu Wang, Mingxia Wu, Chanjun Sun, Han Liu, Hui Liu, Hong Ge

**Affiliations:** ^1^ Department of Radiation Oncology The Affiliated Cancer Hospital of Zhengzhou University Zhengzhou China; ^2^ HNHC Key Laboratory of Radiation Oncology for Henan Cancer Hospital Zhengzhou China; ^3^ Department of pathology and Pathophysiology School of Basic Medical Sciences College of Medicine Zhengzhou University Zhengzhou China

**Keywords:** adverse effect, Electron FLASH, scrotal lesions, extramammary Paget disease, FLASH radiotherapy, ultra‐high dose rate

## Abstract

**Objective:**

Ultrahigh‐dose‐rate radiotherapy (FLASH‐RT) has been shown to reduce radiation‐induced normal tissue injury in preclinical studies. Here, we describe the first patient worldwide to receive fractionated FLASH‐RT.

**Methods:**

An elderly male patient was diagnosed with extramammary Paget disease of the scrotum along with multiple lymph node and bone metastases. After 6 cycles of chemotherapy, the disease progressed in the scrotum. The scrotal lesions were evaluated for FLASH‐RT. 9 MeV electron FLASH‐RT was performed at a dose rate of 120 Gy/s. The field size was 5 cm × 5 cm, and the prescribed dose was 40 Gy in five fractions. Dosimetric verification was performed daily. Treatment response was evaluated at 3‐month post FLASH‐RT, and normal tissue toxicity was assessed from the beginning of FLASH‐RT to 12‐month post‐irradiation.

**Results:**

A 1‐year follow‐up was achieved for scrotal lesions treated with electron FLASH‐RT in patients with extramammary Paget disease. Electron FLASH‐RT was safe, and treatment‐related adverse events were mild. Transient skin toxicity occurred 2–5 weeks post FLASH‐RT, and exudation and burning pain were significantly alleviated. A complete response was achieved 2–3 months post FLASH‐RT, and the tumor lesions were covered by the normal epithelium of the scrotum. At the end of the one‐year follow‐up, the tumor lesions continued to respond, and one‐year survival was achieved without additional injury to the irradiated areas after FLASH‐RT.

**Conclusion:**

Fractionated electron FLASH‐RT is feasible and safe for the treatment of extramammary Paget disease of the scrotum. Our findings support further exploration of electron FLASH‐RT in patients with skin tumors.

## Graphical abstract

An elderly male patient with extramammary Paget disease of the scrotum and multiple metastases was treated with fractionated FLASH‐RT (40 Gy in 5 fractions) in the scrotal lesion.

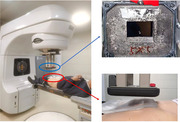

## 1 INTRODUCTION

Extramammary Paget disease is a rare neoplasm of the skin that often involves apocrine gland‐bearing locations including the perianal area and the vulva.^1^ Despite the low incidence, the disease exhibits frequent recurrence and metastatic potential in elderly patients.^2^ Evidence‐based clinical guidelines have been established for extramammary Paget disease, and surgical resection was recommended for operable and localized disease.^3^ Other local therapy strategies such as radiotherapy have been considered an important component in the management of extramammary Paget disease.^4^

Over the past decades, conventional dose‐rate radiotherapy (CONV‐RT) has been delivered in fractions to maximize the destruction of malignant cells while minimizing damage to healthy tissues.^5^ Technological innovations in radiation oncology have led to the development of precision radiotherapy.^6^ Both safety and dose distributions have been improved in CONV‐RT.^7^ However, the overall clinical response rate of CONV‐RT remains suboptimal. Escalation of radiation doses to cancer cells is a promising method to sterilize any tumor and achieve nearly 100% tumor control; however, damage to the surrounding healthy tissue is a key challenge for the delivery of high‐dose radiation.^8^

FLASH‐RT is radiation treatment delivered at ultra‐high dose rates (mean dose rate ≥ 40 Gy/s) compared to CONV‐RT (usually ≤ 10 Gy/min).^9^ The radiation dose can be delivered almost instantaneously in milliseconds with FLASH‐RT, and this dose rate is believed to cause normal tissue sparing without compromising the antitumor efficacy, also known as the FLASH effect.^10^ In recent years, the FLASH effect has been reported in various animal models with different types of irradiations including electron, photon, and proton.^10^, ^11^, ^12^ Although the mechanisms of FLASH‐RT in alleviating normal tissue damage are still under investigation, oxygen depletion, peroxyl radical recombination, and immune cell protection are thought to be essential for the FLASH effect.^9^ This dramatic increase in the differential effect between tumors and normal tissues has accelerated its clinical translation.

In 2019, Lausanne University Hospital performed electron FLASH‐RT in a patient with multi‐resistant CD30+ T‐cell cutaneous lymphoma and found a favorable outcome in this case, both on normal skin and the tumor; the results indicated that electron FLASH‐RT is feasible and safe.^13^, ^14^ Furthermore, the Cincinnati Children's Hospital Medical Center reported the feasibility and safety of proton FLASH‐RT in patients with extremity bone metastases in the FAST‐01 clinical trial.^15^ These promising findings supported the further evaluation of FLASH‐RT in patients with cancer.

The aim of any cancer treatment is to maximize the Therapeutic Index (ratio of tumor control probability to normal tissue complication probability). Jumping directly into single‐dose FLASH‐RT for all cancers is risky. The use of a fractionated schedule is a more cautious and familiar approach for clinicians. Fractionated FLASH‐RT could potentially push this index to unprecedented heights. Each fraction delivers a powerful, tissue‐sparing blow to the tumor, and repeating this process cumulatively helps eradicate the tumor while providing healthy tissue with the best possible chance to recover.^16^

In this study, we evaluated the efficacy and toxicity of fractionated electron FLASH‐RT in a patient with extramammary Paget's disease of the scrotum.

## 2 METHODS

### 2.1 Patient characteristics

**FIGURE 1:**
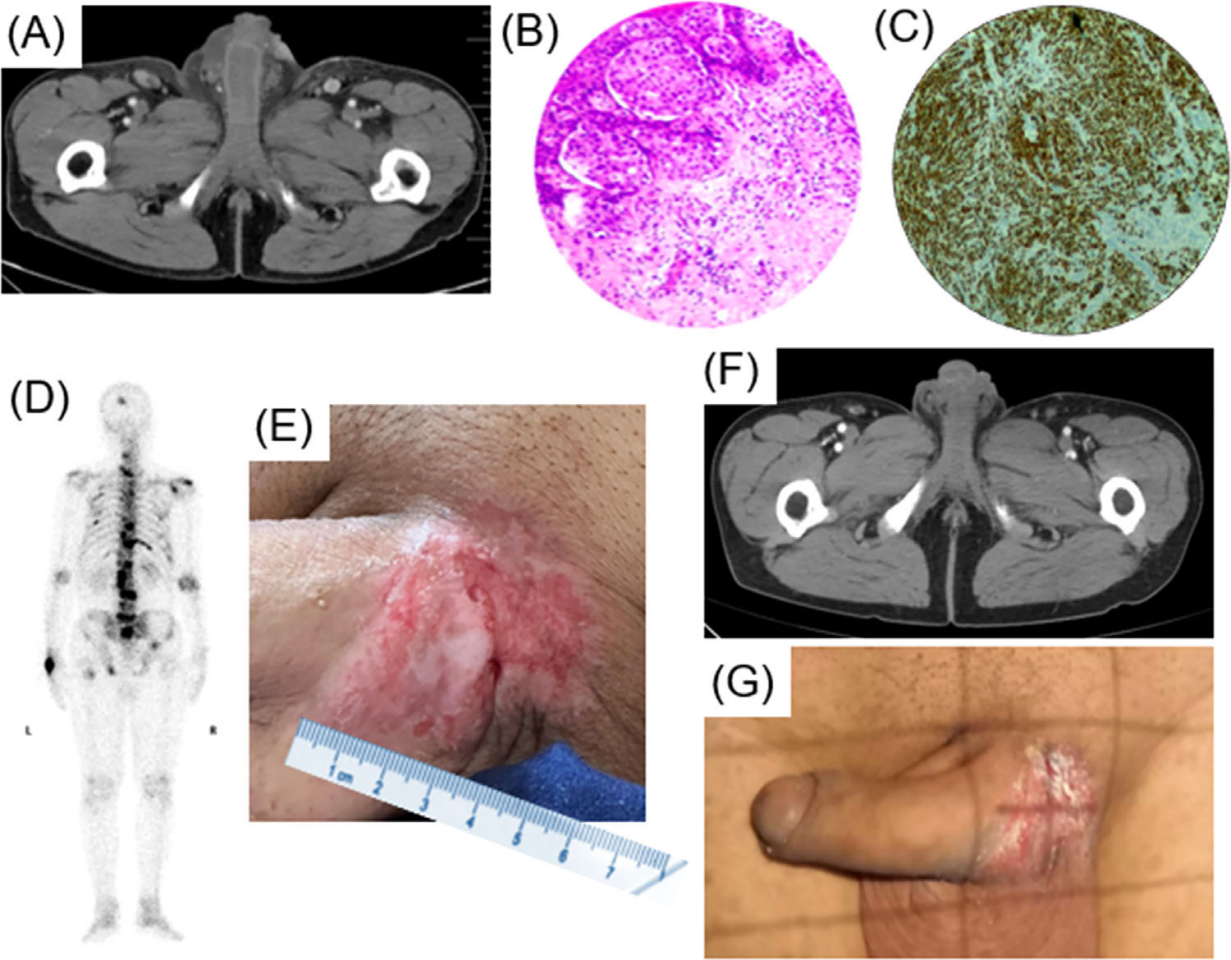
Imaging data and scrotum lesions of the included patient. (A) Pretreatment CT images (July 2022); (B) Representative images of hematoxylin & eosin staining (December 2022); (C) Immunohistochemistry of CK7 (December 2022); (D) Pretreatment Emission Computed Tomography images (December 2022); (E) The patient ’s scrotum lesions (July 2023); (F) CT images after 6 cycles of chemotherapy (July 2023); (G) Radiation simulation. (Fig. 1A and 1F were previously published in Journal of Zhengzhou University (Medical Sciences), and reprinted permission was approved).

The first patient described was reported in our previous publication.^17^ He was a 68‐year‐old patient with extramammary Paget disease. In July 2022, the patient developed scrotal nodules without medical intervention (Fig. 1A). A scrotal nodule biopsy of the lesions was performed in December 2022. The patient was diagnosed with extramammary Paget disease of the scrotum (Fig. 1B). Immunohistochemistry showed: CK7 (+), P40 (‐), P63 (‐), S‐100 (‐), SOX‐10 (‐), P16 (+), P53 (weak +), Ki‐67 (+70 %) (Fig. 1C). Computed tomography revealed multiple lymph node and bone metastases (Fig. 1D). The patient received six cycles of chemotherapy, including cyclophosphamide (1000 mg) and epirubicin (150 mg). CT imaging revealed that the metastatic lymph nodes were smaller. However, in July 2023, erythema, erosion, ulcers, exudation, and burning pain gradually appeared on the skin (Fig. 1E). A CT scan demonstrated that the scrotal nodules were larger than before and that the metastatic lymph nodes were unchanged (Fig. 1F). Chemotherapy did not eradicate cancer cells in the scrotal lesion. Moreover, ulceration and exudation were evident, accompanied by itching and pain in the scrotum, which seriously affected the patient's quality of life. Considering that the tumor location was superficial and the scrotum was fragile compared with other skin areas, the normal tissues surrounding the tumor might tolerate CONV‐RT poorly, which is not conducive to eradicating tumor cells.^18^, ^19^ According to preclinical results, clinical data, and international experience, this patient was considered more suitable for electron FLASH‐RT compared with CONV‐RT.^13^, ^16^ The patient was carefully evaluated by the FLASH‐RT group of the Affiliated Cancer Hospital of Zhengzhou University. The FLASH‐RT group consisted of four senior radiation oncologists, three physicists, and one oncologist. To ensure the same therapeutic effect, FLASH‐RT was proposed to reduce the side effects on the scrotum and improve the quality of life. The patient agreed to undergo FLASH‐RT. After obtaining approval from the Institutional Review Board of the Affiliated Cancer Hospital of Zhengzhou University, the patient signed an informed consent form. The radiation simulation was conducted prior to FLASH‐RT (Fig. 1G).

### 2.2 Interventions (FLASH‐RT)

FLASH‐RT was delivered using 9 MeV electrons. The parameters are listed in Supplementary Table 1. Details of the linear accelerator used for FLASH‐RT, including the profiles and dose distribution curves along the beam field direction, have been reported previously.^20^ All parameters were measured and certified by the China Institute of Atomic Energy. The total radiation dose delivered to the tumors was 40 Gy in five fractions (biologically effective dose (BED_10_) = 72 Gy). This dose was applied because BED_10_ greater than or equal to 60 Gy has been associated with excellent overall cure rates for extramammary Paget disease.^4^ Dosimetric checks were conducted before, after, and during FLASH‐RT. The dose distribution of FLASH‐RT was measured using both alanine pellets and the Gafchromic HD‐V2 Film.

### 2.3 Follow‐up

Radiation‐induced adverse events were assessed according to the National Cancer Institute Common Terminology Criteria for Adverse Events (NCI‐CTCAE v5.0). Acute toxicity was monitored and recorded weekly for the first three months. Late toxicity was evaluated and recorded. Treatment response was assessed using the Response Evaluation Criteria in Solid Tumors (RECIST) (v1.1).^21^ Patient follow‐up was typically scheduled in the clinic with physical examinations, CT imaging, and routine laboratory tests.

## 3 RESULTS

### 3.1 FLASH‐RT delivery

**FIGURE 2:**
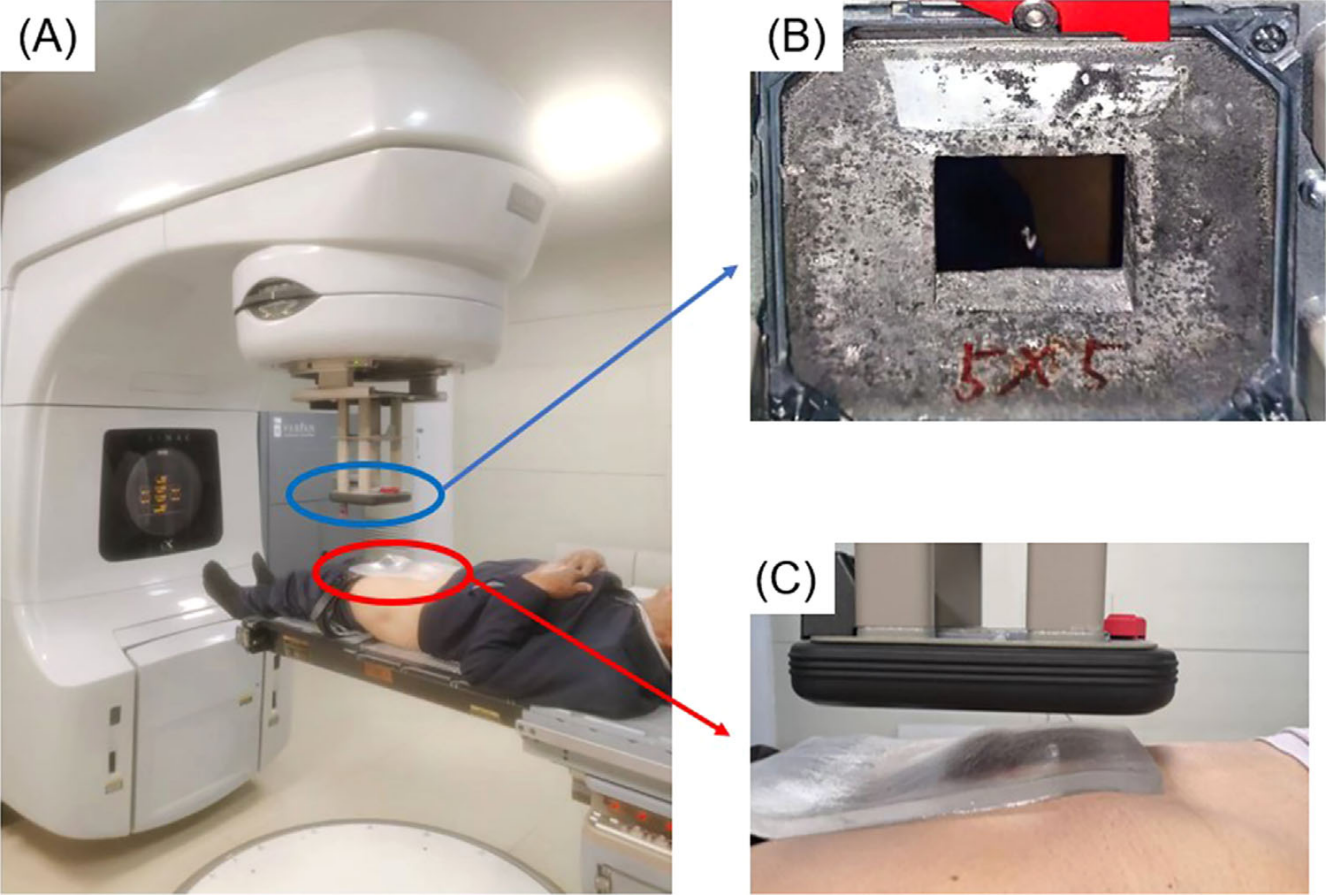
The implementation of FLASH‐RT. (A) Irradiation field setting; (B) Shielding with lead (Blue cycle in Fig. 2A) (C) A bolus was used during FLASH‐RT (Red cycle in Fig. 2A). (Fig. 2B and 2C were previously published in Journal of Zhengzhou University (Medical Sciences), and reprinted permission was approved).

The irradiation field measured 5 cm ×5 cm (Fig. 2A). The surrounding normal tissues were shielded with lead (Fig. 2B). To increase the dose to the tumor area, a 1‐cm bolus was used during treatment (Fig. 2C). The patient received 8 Gy of radiation delivered in < 100 ms per fraction. Dose calibration was performed before, during, and after the treatment. The measured doses were 788.67±10.9, 810.67±5.3, 793.06±7.8, 805.33±6.2 and 784.33±7.3 cGy for the five fractions. The average dose rate was approximately 120 Gy/s.

### 3.2 Acute adverse effects

**FIGURE 3:**
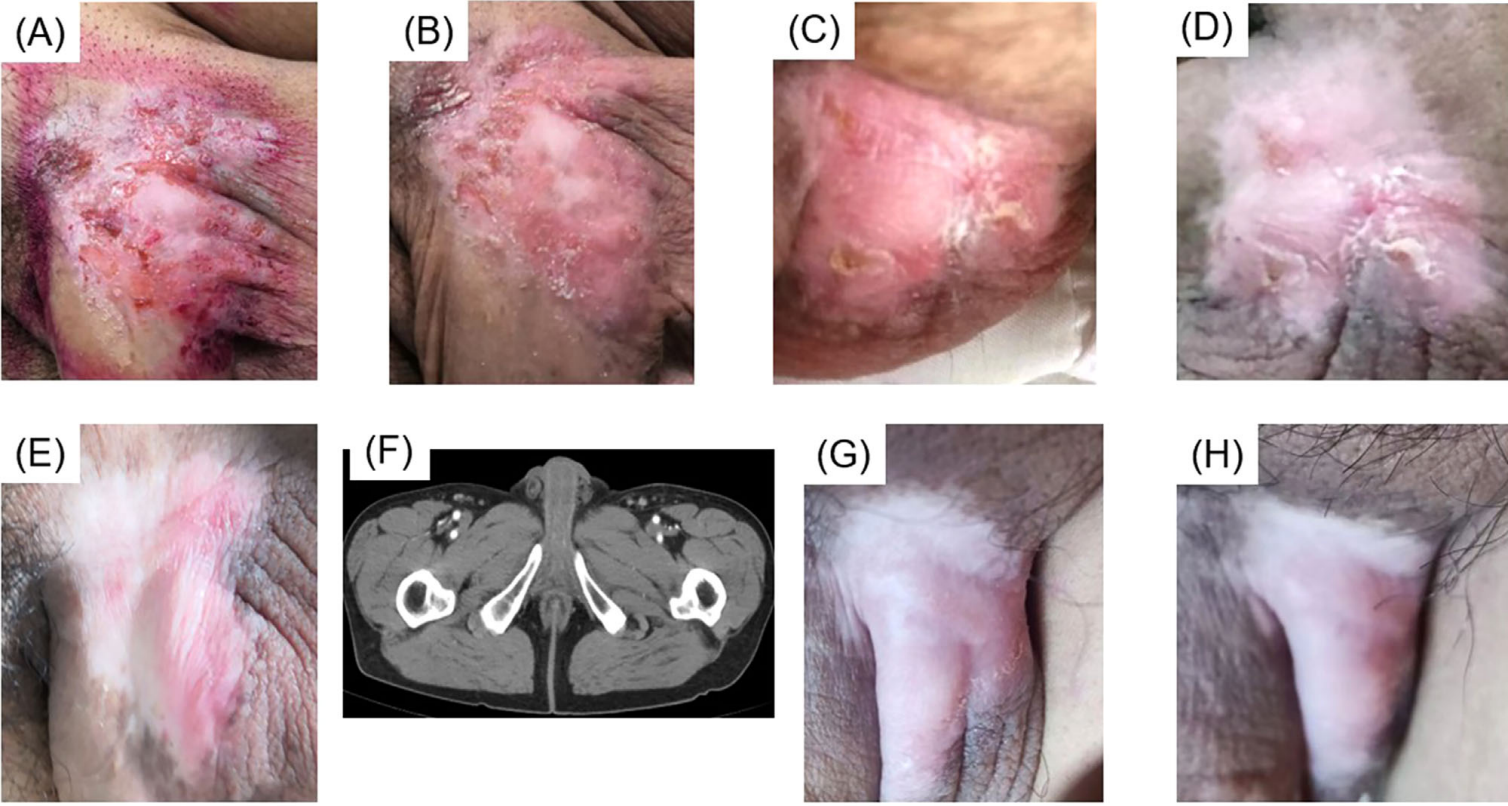
The grade of acute radiation‐induced adverse effects and tumor control after the administration of FLASH‐RT. (A) 1‐week post irradiation; (B) 3‐weeks post irradiation; (C) 6‐weeks post‐irradiation; (D) 2‐months post‐irradiation; (E) 3‐months post‐irradiation; (F) CT images of 3‐months post‐irradiation; (G) 6‐months post‐irradiation; (H) 12‐months post‐irradiation. (Fig. 3A, 3B, and 3E were previously published in Journal of Zhengzhou University (Medical Sciences), and reprinted permission was approved).

Within the first week after FLASH‐RT, there were no significant changes in the normal tissues surrounding the tumor (NCI‐CTCAE, grade 0, Fig. 3A). At 2–5 weeks post‐irradiation, dark erythema was observed on the normal skin surrounding the tumor, combined with mild edema, dry peeling, and mild tenderness; there was no wet peeling, bleeding, ulcer, or necrosis. These findings indicated mild radiation dermatitis. The third week was the peak period of adverse skin reactions (NCI‐CTCAE, grades 1–2; Fig. 3B). After 6 weeks, the symptoms gradually decreased and resolved (Fig. 3C–3E). At the 6‐ and 12‐month follow‐up, only pubic hair loss was observed, and visual inspection indicated no other treatment‐related adverse events (Fig. 3G and 3H).

### 3.3 Treatment efficacy evaluation

Both congestion and exudation were similar to those at pretreatment within the first week after FLASH‐RT, and redness and mild swelling were observed (Fig. 3A). After 2–4 weeks, the exudation gradually increased, and the exudative and ulcerative lesions intermittently solidified and formed scabs. The tumor tissues began to shrink, and granulation tissue began to fill the ulcer site (Fig. 3B). At week 6, the tumor tissues shrank significantly, and the scab gradually sloughed off, the normal tissue was repaired, the skin surface became rough, and the skin color became bright red with itching (Fig. 3C). From week 8, a complete response was achieved, and the scabs detached. The tumor area was replaced by normal epithelial tissue with a hard texture, poor elasticity, and reduced itching and pain (Fig. 3D). Three months after the FLASH‐RT, the surface of the entire irradiation field was smoother and more delicate. Normal tissue repair was complete, and adverse effects—including itching and pain—had disappeared (Fig. 3E). CT imaging revealed complete eradication of the scrotal nodules (Fig. 3F). At the last follow‐up, 1‐year survival was achieved without recurrence of the irradiated lesions after FLASH‐RT (Fig. 3G and 3H).

## 4 DISCUSSION

This is the first study to report the feasibility and safety of fractionated electron FLASH‐RT. In the present case of extramammary Paget disease within the scrotum, the adverse effects on normal tissues were mild (grade 2 or 1). After treatment with FLASH‐RT, a complete response was maintained until the last follow‐up; symptoms including erythema, erosion, ulcers, and exudation were effectively controlled, and the patient's quality of life was significantly improved.

Extramammary Paget disease is a slow‐growing intraepithelial malignant neoplasm that mainly develops in areas with apocrine sweat glands and typically affects elderly patients. Cases that occur primarily in the scrotum are extremely rare. Although most early extramammary Paget disease lesions are indolent, individuals with metastatic extramammary Paget disease have poor survival because of disease progression and lack of effective systemic treatment. In the present study, the patient was diagnosed with metastatic extramammary Paget disease of the scrotum, and systematic chemotherapy was administered. Although a partial response was achieved for the metastatic lesions, the drugs failed to control the disease in the scrotum. Typical symptoms of the scrotum include itching and burning pain. As the disease progressed, eczematoid, crusted, ulcerated, papillary lesions were observed. These results indicate the refractory nature of extramammary Paget disease of the scrotum.

Previous studies have shown that palliative radiotherapy can be used to improve the local control rate of tumors and relieve the series of symptoms caused by extramammary Paget disease of the scrotum.^4^ In our case, a fractionated FLASH‐RT modality (8 Gy × 5 fractions) with a BED of 72 Gy was delivered to achieve equivalent antitumor efficacy while reducing radiation‐induced injury compared to CONV‐RT. Three months later, the eczematoid tumors in the scrotum were completely eradicated and symptoms, including itching and burning pain, were markedly relieved. Our results showed that a BED > 60 Gy was associated with an optimal radiation response and that fractionated FLASH‐RT is safe and feasible for extramammary Paget disease of the scrotum.

Indeed, the Group from Lausanne University Hospital used a single dose of 15 Gy electron FLASH‐RT (average dose rate 166 Gy/s, delivered in less than 100 ms) to treat a patient with cutaneous lymphoma; a maximal grade 1 adverse effect (epithelitis and edema) was observed between days 10 and 44; complete tumor response was achieved 36 days after FLASH‐RT and without recurrence thereafter.^13^ At present, there are two ongoing clinical trials performed by the group using electron FLASH‐RT for skin cancer. One was a single‐center phase I, first‐in‐human, dose‐escalation study of FLASH therapy (NCT04986696) in patients with skin melanoma metastases that progressed locally despite systemic treatment (seven dose levels: 22 Gy, 24 Gy, 26 Gy, 28 Gy, 30 Gy, 32 Gy, and 34 Gy). The other was a Phase II study of FLASH‐RT (NCT05724875) versus conventional RT in patients with localized cutaneous squamous cell carcinoma or basal cell carcinoma (a single dose of 22 Gy for T1 lesions and a 5 × 6 Gy fractionated dose for T2 lesions). Furthermore, our group designed a Phase I study (ChiCTR2400080935) to describe and evaluate the toxicity and efficacy of FLASH‐RT in patients with localized non‐melanoma skin cancer requiring radiation treatment.^22^

In addition, a group from the Cincinnati Children's Proton Therapy Center performed proton FLASH‐RT (NCT04592887) for the palliative treatment of painful bone metastases; a total of 10 patients were included, and a single dose of 8 Gy was delivered.^15^ Acute treatment‐related toxicities of FLASH‐RT included grade 1 edema, erythema, fatigue, pruritus, skin hyperpigmentation, and grade 2 extremity pain. Long‐term toxicity of grade 1 skin discoloration was observed in only one patient. Overall, the adverse events associated with proton FLASH‐RT were mild and consistent with those associated with CONV‐RT. This group is currently conducting another clinical trial to assess the toxicity of FLASH‐RT (NCT05524064) and pain relief in patients with painful thoracic bone metastasis. Undoubtedly, FLASH‐RT is a novel milestone in the field of radiotherapy.

Dose and fractionation schedules for FLASH‐RT are still under investigation. In general, the incidence of radiation damage to healthy tissues directly correlates with the amount of radiation exposure.^23^ In CONV‐RT, fractional irradiation is applied to maximize the destruction of tumor cells while minimizing injury to normal tissues.^24^ Current evidence for the influence of dose rate on radiation‐related injury is encouraging, and FLASH‐RT is a promising technique to reduce damage to normal tissues and organs with a single high‐dose prescription.^25^ However, it is unclear whether the fractionation schemes and treatment modalities utilized in CONV‐RT are also applicable to FLASH‐RT. Böhlen et al. demonstrated that the FLASH effect was tissue‐specific, and the normal tissue sparing effect was much more significant with an increase in irradiation dose.^26^ The FLASH effect was insignificant compared with CONV‐RT in the first patient treated with FLASH‐RT at Lausanne University Hospital.^13^ In a phase 3 animal trial, severe late radiation injury was observed in cats combined with primary squamous cell carcinoma.^27^ This indicated the potential limitations of delivering FLASH‐RT in a single high dose. Maity et al. suggested that the late radiation‐associated side effects of FLASH‐RT were correlated with a large radiation field.^28^ The group from Lausanne University Hospital suggested that FLASH‐RT‐associated radiation injury was about 1/3 less than that of CONV‐RT, and a single high‐dose irradiation may conceal the potential protective effect of normal tissues behind FLASH‐RT.^9^ In the meantime, basic research has suggested that a single irradiation dose less than 5 Gy was not enough to trigger the FLASH effect.^29^ Therefore, the dose range within each fraction is vitally important prior to the implementation of fractionated FLASH‐RT in clinical trials. To achieve optimal normal tissue protective effects without attenuating tumor control rates, fractional radiotherapy with a single moderate dose modality may be appropriate for FLASH‐RT.^16^ Because of the unique radiobiological mechanisms of FLASH‐RT, whether BED or equivalent dose in 2‐Gy fractions (EQD2) can be used to evaluate the biological effect of fractional FLASH‐RT is not yet understood, and further investigation is necessary. In addition, published studies have focused primarily on acute radiation‐associated toxicities during and after FLASH‐RT;^30^ long‐term follow‐up of late adverse effects is needed. The detailed mechanism of the late side effects of FLASH‐RT remains to be fully elucidated.

The integrated process of FLASH‐RT is complex and involves an understanding of the principles of radiochemistry, medical physics, radiation biology, treatment planning, radiation dosimetry, simulation, radiation safety and protection to ensure accurate and safe delivery of treatment.^13^, ^15^ It is recommended that the FLASH‐RT quality control and quality assurance report contain the following parameters: beam energy, pulse repetition rate, duty cycle, temporal pulse structure, beam intensity, cumulative dose per delivery, dose per pulse, instantaneous dose rate, average dose rate per beam, mean dose rate per fraction, dose distribution per beam and per fraction.^31^ Because the instantaneous dose rate of FLASH‐RT is significantly higher than that of CONV‐RT, ionization chambers and semiconductors will achieve saturation during FLASH‐RT.^32^, ^33^ Alanine dosimeters, thermoluminescence dosimeters, Gafchromic^TM^ EBT films, and photoluminescent dosimeters have been utilized to measure the dose rate of FLASH‐RT.^34^, ^35^ The diamond detector exhibited considerable linearity of dose response to both electron and photon FLASH‐RT, and could provide a relatively rapid and accurate dosimetry.^36^ In addition, image‐guided FLASH‐RT can be considered to ensure real‐time tracking of tumors and improve dose delivery.

FLASH‐RT is different from CONV‐RT in that it spares normal tissues. Whether FLASH‐RT has similar radiation sensitivity to CONV‐RT remains unclear. Sensitivity to FLASH‐RT may facilitate further screening of patients eligible for FLASH‐RT. In a phase 3 animal trial of primary nasal squamous cell carcinoma in cats, one cat in the FLASH‐RT group experienced disease progression 1‐year post‐irradiation, while no tumor progression was observed in the CONV‐RT group.^27^ The results suggested that individual sensitivity to radiation may lead to different treatment responses. A previous bioinformatics analysis of human acute lymphoblastic leukemia showed that genetic factors may be involved in regulating the sensitivity of FLASH‐RT.^37^ However, the sample size was small, and further exploration is needed.

The present study has some limitations. The description of the chemotherapy regimen does not adhere to standard formatting conventions. A crucial scientific question for FLASH‐RT is whether standard BED models derived from CONV‐RT are directly applicable. Additionally, since the assessment of adverse events was based solely on visual inspection and symptomatic evaluation without histological confirmation, more objective measures (e.g., androgen levels) are needed.

## 5 CONCLUSIONS

In conclusion, the present study evaluated the toxicity of electron FLASH‐RT for the treatment of extramammary Paget's disease of the scrotum. Our results demonstrate that FLASH‐RT is safe and feasible and warrant further investigation.

## AUTHOR CONTRIBUTIONS

HL and CLY were responsible for study design and interpretation. All authors contributed to the writing of the manuscript. Ma LJ, Mao RH, Lei HC, and Peng C contributed to dosimetric data collection. Zhang YP, Xu M, Wang C, Wang YW, Wu MX, Sun CJ, and Liu H contributed to clinicopathological data collection. Liu H, and Ge H contributed to the study design and critical manuscript revisions. All authors have read and reviewed the manuscript and approved its submission.

## CONFLICT OF INTEREST STATEMENT

The copyright was acquired from the Journal of Zhengzhou University (Medical Sciences). Among the four senior radiation oncologists, Dr. Luo has conducted postdoctoral research on FLASH‐RT at Lausanne University Hospital. The authors declare that they have no competing financial interests or personal relationships that may have influenced the work reported in this study.

## ETHICAL APPROVAL

This study was approved by the Institutional Review Board of the Affiliated Cancer Hospital of Zhengzhou University (2023‐KY‐0148‐003).

## Supporting information



Supporting Information
