# Supplementary material for: One‐year follow‐up after fractionated ultra‐high‐dose‐rate FLASH radiotherapy in patient with extramammary Paget disease of the scrotum
Source: Precis Radiat Oncol. 2025 Dec 26;10(2):120–6. doi: 10.1002/pro6.70045 (PMC13292164; doi:10.1002/pro6.70045)
Supplement: Supplementary file 1 — Supporting Information [file PRO6-10-120-s001.docx]

Table S1.

The parameters of electron FLASH-RT from modified Varian 23CX linear accelerator.

| Irradiation modality | Energy (MeV) | Average dose rate (Gy/s) | Pulse number | Frequency  (Hz) | Pulse width  (µs) | Dose per pulse (Gy) | Instantanous dose rate (Gy/s) | SSD  (cm) |
| --- | --- | --- | --- | --- | --- | --- | --- | --- |
| FLASH-RT | 9 | 120 | 360 | 360 | 4.0 | 0.33 | 82500 | 100 |

Abbreviation: CONV-RT: conventional radiotherapy; FLASH-RT, FLASH radiotherapy; SSD: source‐to‐surface distance.
